# Supramolecular Partners: Precision On/Off Neuromuscular Blockage for Prolonged Surgeries

**DOI:** 10.1021/acs.jmedchem.5c00557

**Published:** 2025-03-06

**Authors:** Ziyi Wang, Hang Yin, Ruibing Wang

**Affiliations:** State Key Laboratory of Quality Research in Chinese Medicine, Institute of Chinese Medical Sciences, & Department of Pharmaceutical Sciences, Faculty of Health Sciences, University of Macau, Taipa, Macau SAR 999078, China

## Abstract

The development of a long-acting neuromuscular blocking agent and its on-demand reversal is highly desired for prolonged surgeries. This viewpoint discussed the discovery of supramolecular partners, an imidazolium-based macrocycle (YW70271) and acyclic cucurbit[n]uril (WY22051), for ultralong neuromuscular blockade and rapid reversal.

Controllable anesthesia is crucial for prolonged surgeries to ensure patient safety, maintain optimal surgical conditions, and improve overall outcomes. Therefore, the development of long-acting anesthetic and strategy for rapid on-demand reversal is highly desired for prolonged surgeries.^1^ In a Featured Article in this issue of the *Journal of Medicinal Chemistry*, Li et al. developed supramolecular partners: an imidazolium-based macrocycle (YW70271) and an acyclic cucurbit[n]uril derivative (WY22051), as an ultralong-acting neuromuscular blocking agent with rapid on-demand reversal (Figure 1). Through structure–activity optimization, YW70271 achieved an unprecedented neuromuscular blockade duration of 217 min (surpassing the clinical long-acting agent pancuronium at 20.5 min) and a rapid onset of 20.5 s (faster than intermediate-acting cisatracurium). By introducing WY22051 as a supramolecular antagonist, the time for TOF recovery to 90% was reduced to 19 s (outperforming sugammadex-mediated reversal of rocuronium), enabling precise, real-time control of anesthesia for prolonged surgical interventions.^2^

**Figure 1:**
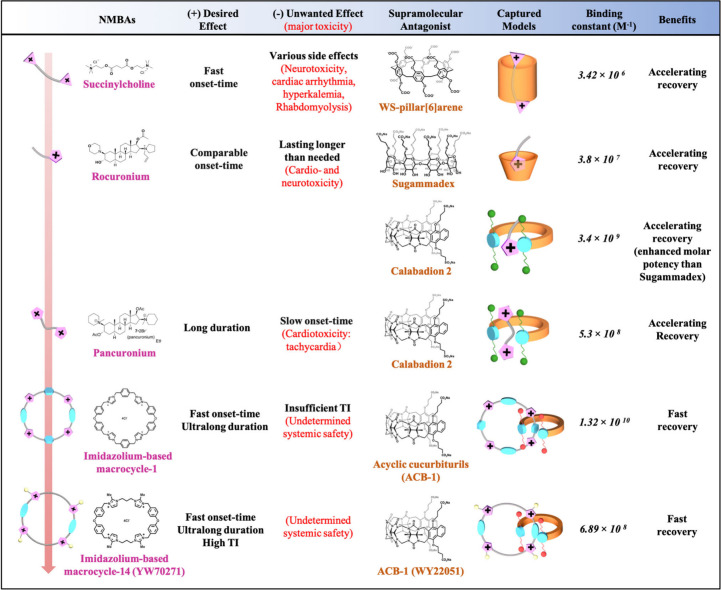
Development of representative quaternary ammonium groups-based NMBAs and their supramolecular antagonists, highlighting the benefits, drawbacks, and major advancements achieved of NMBAs and corresponding supramolecular partners, respectively.

Neuromuscular blocking agents (NMBAs) remain a cornerstone of modern anesthesia for intraoperative muscle relaxation; they are broadly classified as depolarizing (e.g., succinylcholine) and nondepolarizing agents (e.g., rocuronium, cisatracurium), with over 400 million annual administrations worldwide. Depolarizing agents, such as succinylcholine, directly activate acetylcholine receptor, offering rapid onset muscle relaxation, while their association with hyperkalemia and malignant hyperthermia limits its clinical use. On the other hand, nondepolarizing NMBAs work through competitive inhibition of acetylcholine receptors and are classified into the short, intermediate and long-acting agents according to the duration time: 1) Short-acting agents are struggling (e.g., gantacurium analogs) with metabolism independent of organ function. 2) Intermediate-acting NMBAs, such as rocuronium and cisatracurium, dominate clinical practice due to improved safety and compatibility with antagonists. However, their limited duration in prolonged surgeries necessitates repetitive dosing and careful monitoring. 3) Long-acting agents such as pancuronium face declining use due to irreversibility and prolonged recovery, underscoring the unmet need for agents combining extended efficacy with prompt, on-demand reversibility.^1^

Compelling evidence underscores the high incidence of postoperative residual neuromuscular blockade (RNMB) when NMBAs are not pharmacologically reversed. Studies reveal alarmingly high rates of inadequate recovery (train-of-four ratios, TOFr < 0.7–0.9) in patients receiving vecuronium (42%), cisatracurium (57%), rocuronium (44–89% in elderly cohorts), or intermediate-acting agents (45%) without reversal, increasing risks of aspiration and pneumonia. The ideal NMBA should not only guarantees the nonaccumulative and nonadverse biocompatibility, but also possess fast onset, adequate duration, rapid offset and reversibility by antagonist. Current pharmacological reversal primarily relies on carbamate moiety, exemplified by neostigmine, an acetylcholinesterase inhibitor, which has remained the standard reversal agent despite its limitations, including residual muscle weakness, increased risk of postoperative respiratory complications, and inadequate efficacy in reversing profound neuromuscular blockade. The advancement of modern anesthesia has spurred growing interest in developing paired compounds as “on–off switches” to achieve precision control of neuromuscular blockade. Emerging agents such as gantacurium, CW002, and CW011 are under preclinical investigation, showing promising features like rapid onset and reversibility through mechanisms involving edrophonium or l-cystine. However, challenges persist, including transient duration of action, delayed recovery profiles, and organ-dependent metabolic pathways that may limit clinical utility. These developments underscore the critical need for tailored reversal systems to match evolving NMBA pharmacology in precision anesthesia paradigms.^1^

In recent years, macrocycle-based supramolecular antidotes have emerged as new agents to alleviate biomolecules’ toxicity, side effects, and even anesthesia effect, showing great potential in improving NMBA management (Figure 1),^3^ especially since the approval of sugammadex in 2015. Sugammadex, a cyclodextrin derivative, enables rapid and dependable reversal of neuromuscular blockade through host–guest encapsulation, prompting the controllability of modern anesthesia. This breakthrough also paved the way for the development of supramolecular hosts such as carboxylatopillar[6]arene (WP[6]) and cucurbit[n]urils (calabadions, acyclic cucurbiturils) as potential antidotes for a wide range of toxic molecules including NMBAs.^4−6^ Notably, Calabadion-2, exhibits an 89-fold increase in binding affinity for rocuronium (K_a_ = 3.4 × 10^9^ M^–1^) compared to sugammadex, allowing dynamic dose-dependent behaviors in reversing both steroidal and benzylisoquinoline NMBAs with high biocompatibility.^6^ Thus, they have the potential to emerge as the next generation of reversal agents, extending their applicability beyond perioperative settings.

The postsynaptic nicotinic acetylcholine receptor (nAChR) and acetylcholinesterase share structural homology in their anionic binding regions, both requiring quaternary ammonium groups for ligand interaction. This principle underpins the design of NMBAs: succinylcholine, a depolarizing agent, structurally mimics two covalently linked acetylcholine molecules to simultaneously engage both nAChR binding sites required for ion channel activation.^7^ Similarly, classical nondepolarizing agents like pancuronium and atracurium adopt bis-quaternary ammonium architectures, with two cationic centers spaced 10–12 carbons apart to optimize receptor affinity and potency.^8^ In contrast, monoquaternary aminosteroid NMBAs (e.g., rocuronium) exhibit reduced binding avidity due to single-site engagement, yet benefit from faster onset kinetics through enhanced tissue diffusion. These structure–activity relationships highlight the delicate balance between molecular geometry, charge distribution, and pharmacokinetic performance in NMBA development.

Based on the previous structure–activity relationship, Li and co-workers have reported pseudo[2]catenane partners, composed of imidazolium-based macrocycles (IMCs) and acyclic cucurbit[n]urils (ACBs), for prolonged neuromuscular blockade and on-demand, rapid reversal.^9^ By investigating structure–property studies, they concluded that the tetracationic nature of IMCs, characterized by quaternary ammonium groups, allows for multivalent interactions with nicotinic cholinergic receptors. Meanwhile, benefiting from the ammonium units, IMCs could be encapsulated by ACB with a high binding affinity (K_a_ > 10^9^ M^–1^), presenting ACB as an effective antagonist capable of rapid reversal. Notably, prototype IMCs demonstrated human-equivalent blockade durations of 158–442 min at 2–3 × ED_90_ in rat models, with ACB-mediated recovery at a rate 3.8-fold faster than sugammadex, indicating a substantial advancement though the therapeutic index (TI) remains unsatisfactory.

Building upon previous research on IMCs, Li’s team systematically optimized the IMC structures to overcome concerns surrounding limited TI and slow onset time, with the aim to achieve an ultralong-acting neuromuscular blockade while preserving the rapid on-demand reversibility by ACBs. Accordingly, they identified key efficacy determinants, including the importance of cationic moieties, the macrocycle backbones, and aliphatic substituents. Among these candidates, IMC-7 emerged as a primary contender, exhibiting a remarkable TI of 100% and ED_90_ of 0.016 mg/kg—15 times lower than pancuronium, the clinically long-acting NMBA. Expanding on the success of IMC-7, the researchers introduced structural modifications by substituting meta-phenylene units with aliphatic chains to create IMC-13–19, and evaluated benzimidazolium-incorporated analogue IMC-20, propylene-derived smaller macrocycles IMC-21 and IMC-22, and pyridinium-incorporated derivative PMC-1. Among these varied compounds, IMC-14 (YW70271) distinguished itself with exceptional potency, demonstrating an ED_90_ of 0.016 mg/kg and rapid reversibility within 19 s using ACB-1 (WY22051), compared to 72 s required for sugammadex-rocuronium reversal. This rigorous structural screening approach successfully balanced receptor binding kinetics with metabolic stability, effectively addressing previous concerns related to therapeutic index and onset time without compromising the duration of action. These results mark a significant advancement in the development of long-acting NMBAs and its on-demand reversal, offering a therapeutic profile that combines prolonged surgical efficacy with swift reversibility. The optimization framework outlined in this study not only discerns a prospective clinical candidate but also furnishes the structure–property relationship in NMBAs, providing crucial insights into future studies of nicotinic receptor blockers. Through direct comparison with established agents during the development phase, this study minimized the divide between preclinical exploration and clinical integration.^2^

Despite these advances, translational challenges persist. Interspecies pharmacodynamic disparities—evident in rodent-to-human differences in ED_90_, TOFr, and onset times—necessitate validation in higher-order species prior to clinical trials. Adverse effects, particularly anaphylaxis, occasionally occur with NMBAs. Hence, it is crucial to thoroughly monitor allergic reactions and prepare preventive measures related to YW70271 and WY22051. Long-term biosafety assessments of macrocyclic compounds remain imperative, particularly regarding off-target interactions and metabolic byproducts. Besides, scalability of synthesis presents a hurdle, as clinical use of complex macrocycles such as ACBs would require cost-effective, industrial-scale production methods without compromising purity. Furthermore, balancing specificity and broad-spectrum utility remains critical: highly selective agents risk limited applicability, while generalist hosts may incur toxicity.^3^ Nevertheless, the advancement of supramolecular partners (i.e., paired compounds) acting as “on–off switches” for precise regulation of neuromuscular blockade shows significant potential for clinical application.
